# Host population structure impedes reversion to drug sensitivity after discontinuation of treatment

**DOI:** 10.1371/journal.pcbi.1005704

**Published:** 2017-08-21

**Authors:** Jonas I. Liechti, Gabriel E. Leventhal, Sebastian Bonhoeffer

**Affiliations:** 1 Institute for Integrative Biology, ETH Zürich, Zürich, Switzerland; 2 Department of Civil and Environmental Engineering, Massachusetts Institute of Technology (MIT), Cambridge, Massachusetts, United States of America; University of California, Los Angeles, UNITED STATES

## Abstract

Intense use of antibiotics for the treatment of diseases such as tuberculosis, malaria, *Staphylococcus aureus* or gonorrhea has led to rapidly increasing population levels of drug resistance. This has generally necessitated a switch to new drugs and the discontinuation of older ones, after which resistance often only declines slowly or even persists indefinitely. These long-term effects are usually ascribed to low fitness costs of resistance in absence of the drug. Here we show that structure in the host population, in particular heterogeneity in number of contacts, also plays an important role in the reversion dynamics. Host contact structure acts both during the phase of intense treatment, leading to non-random distributions of the resistant strain among the infected population, and after the discontinuation of the drug, by affecting the competition dynamics resulting in a mitigation of fitness advantages. As a consequence, we observe both a lower rate of reversion and a lower probability that reversion to sensitivity on the population level occurs after treatment is stopped. Our simulations show that the impact of heterogeneity in the host structure is maximal in the biologically most plausible parameter range, namely when fitness costs of resistance are small.

## Author summary

The rising levels of drug resistance in many human infections are cause of great concern for public health. There is a repeating pattern of introduction of new drugs, rise of resistance to these drugs, and phasing out ineffective drugs once resistance has become common. With a decreasing rate of drug discovery it is important to study the dynamics of reversion back to sensitivity for drugs that are no longer in use in the host population. While it is known that fitness cost of resistance plays an important role in this reversion process, this study is the first to show that structure in the host population also heavily impacts the reversion dynamics.

## Introduction

The emergence of resistance of infectious pathogens to antimicrobial drugs is a growing concern for public health. The control of many infectious diseases such as tuberculosis, malaria, HIV, or gonorrhea show a recurring historical pattern of the introduction of new potent drugs that initially control the infection efficiently, followed by the subsequent evolution of resistance once the drugs are used widely [1–3]. Over time, a combination of various factors, such as decreases in efficacy, adverse side effects, difficulties in administration, and overall economic costs may lead to changes in treatment guidelines. What generally follows is the successive introduction of novel drugs and the subsequent phasing out of old ones.

Such a pattern of drug use has important consequences for the evolutionary dynamics of resistance. During phases of intense use, the emergence and subsequent spread of resistant strains is driven by the large selective advantage of the resistant over the sensitive strains in presence of treatment [4]. This has been the case in many diseases, such as tuberculosis [5], malaria [6], *Staphylococcus aureus* [7], gonorrhea [8, 9] and HIV [10]. If drug use is discontinued due to changes in treatment guidelines or other factors we generally observe either of two scenarios: One, the resistant strain may decrease in frequency as a result of the change in selective pressure and the population reverts back to high levels of drug sensitivity. For example, in Malawi the clinical efficacy of the antimalarial chloroquine had fallen to low levels because of a high frequency of resistance. But efficacy increased again during the 12 years after the cessation of its use in 1993 from less than 50% to 99% in 2005 [11, 12]. Two, the resistant strain may continue to persist for prolonged periods of time despite substantial reductions in drug use. Examples include the resistance to several antibiotics in *Neisseria gonorrhoeae* [13], streptomycin resistance in tuberculosis [14, 15], sulphonamide and trimethroprim resistance in *Escherichia coli* [16, 17], as well as the persistence of vancomycin resistant enterococci in pigs after the ban of avoparcin in 1995 [18, 19].

Multiple factors have been proposed that might impede reversion back to sensitivity. Firstly, if the reduction in drug use is not substantial enough or only affects a subpopulation of hosts, then the residual use of drugs in the population can sustain the selection for resistance. Secondly, the absolute differences in fitness between resistant and sensitive strains in absence of treatment are generally considerably smaller than in presence of treatment [4]. This asymmetry in fitness differences between the absence and presence of treatment can further be exacerbated by compensatory mutations that alleviate fitness costs associated with resistance mutations [20–22]. Finally, reversion *in vitro* is impeded by genetic interactions between resistance and compensatory mutations. Such interactions can obstruct reversion back to sensitivity, because reverting only the resistance mutation without reverting the compensatory mutation or *vice versa* is associated with a fitness decline in the absence of drugs [23]. However, the obstruction of reversion back to sensitivity due to such fitness valleys is only expected to be relevant in situations where the sensitive strain has to re-emerge *de novo* from the compensated resistant strain by mutation. As changes in treatment guidelines typically occur much before the resistant strain has fixed in the pathogen population across hosts, we expect that the crossing of fitness valleys is likely not relevant for the reversion back to sensitivity in epidemiological scenarios. For malaria in Malawi, for example, the reversion is believed to have occurred through a re-expansion of the susceptible parasite in the population and not through *de novo* back mutation [24].

In the context of antibiotic resistance, Johnsen *et al.* [25] listed further effects that may contribute to the persistence of resistance in absence of drugs. These include selection of other beneficial traits genetically linked to the resistance gene, the role of reacquisition of resistance through horizontal gene transfer and mechanisms preventing plasmid loss. While all of these factors plausibly contribute to obstructing or slowing the reversion process [26], it is difficult to conclusively demonstrate which factors are at play for any particular resistant pathogen population or even to demonstrate that together they are sufficient to explain a slow or absent reversion.

Common to all these factors is the pathogen-centric view with only little attention given to the role of the host. In particular, host contact structure may be another important factor modulating the evolutionary dynamics of resistance. A large body of theory has shown that contact structure profoundly effects epidemiological dynamics [27–32]. Furthermore, Lieberman et al. [33] showed that host structure can affect evolutionary dynamics under a Moran process [34], by modulating the relative importance of selection and random drift. Similarly, both theory and experimentation have established that spatial structure, a specific form of contact structure, influences the evolution of virulence [35–39].

Another element that is absent in the pathogen-centric view is the between-host transmission of resistance. Studies suggest that a resistant strain with only a small fitness deficit in the absence of treatment impacts the overall course of an epidemic in a manner largely independent of its probability of *de novo* emergence [40–42]. Hence, transmission of resistance cannot be neglected and it is particularly important to understand how resistant pathogen strains compete with the wild type throughout the course of an epidemic.

The epidemiological dynamics of wild-type and resistant strains can be seen as a special case of two distinct pathogen strains that are simultaneously spreading in the same host population. Such cases have been studied theoretically for self-limiting dynamics (e.g. SIR-type models), where infected hosts are removed from the population upon recovery, ultimately leading to a depletion of available hosts. Under these dynamics it is possible for two pathogens to both cause an epidemic when spreading sequentially in the same host population, even if infection with the first pathogen confers immunity towards the second [43]. Further studies generalized this finding to cases where the second strain starts to spread simultaneously [44] or with only a small delay [45].

The successful spread of the second pathogen depends on the residual network of susceptible hosts that remains after the first pathogen has spread. In host populations with heterogeneous contact distributions, a host’s likelihood of infection increases with the number of its contacts [32]. This leads to a specific structure of infected hosts in the population and, as a consequence, the residual network left for the second pathogen to spread on does not represent a uniform sub-sample of the entire network [46]. Bansal *et al.* [46] showed that for random networks with fixed average number of contacts an increase in contact heterogeneity leads to a decrease in the epidemic size of the second pathogen.

The concept of residual networks is less clear for non-self-limiting dynamics (e.g. SIS models), where an infected host returns to a susceptible state after recovery. In such models, the mere advantage of spreading more rapidly is less evident as competition arises through co-existence in a continuous epidemic. We have recently shown that in the case of a continuous epidemic heterogeneity in contact structure impedes the invasion of a second fitter pathogen when starting from a single individual in the population [47].

To be able to understand the evolution of resistance in the context of treatment, it is crucial to take into account the two mechanisms of how resistance increases on a population level: either through *de novo* emergence in treated patients or through transmission [48]. A first step was made by Hébert-Dufresne *et al.* [49] who extended models of self-limiting dynamics to incorporate treatment and treatment failure leading to *de novo* emergence of resistance. They showed that even small changes in the ratio of the strains’ fitnesses can drastically affect the total epidemic size.

Here, we propose an epidemiological modelling framework with explicit host contact structure to study non-self-limiting dynamics. This framework allows studying for an endemic disease both the process of resistance emergence during treatment, as well as the subsequent competition dynamics between resistant and sensitive strains after treatment is discontinued. We first describe the general dynamics of a non-self-limiting susceptible-infected-susceptible (SIS) model on a heterogeneous contact network prior to, during and after treatment. Then, we assess the likelihood of reversion to a drug sensitive wild-type population after stopping treatment. Finally, we investigate how the spread of resistance is influenced by the network structure and the colonization of the network by the resistant strain during treatment. Our simulations confirm that contact heterogeneity lowers the probability of reversion back to sensitivity, even when a substantial fraction of the pathogen population is sensitive at the time point when treatment is stopped. Our study reveals that large fitness differences between sensitive and resistant strains result in a non-trivial distribution of sensitive and resistant infections over the network, which in turn influences the reversion dynamics once treatment is stopped. Importantly, the modulating effects of host contact structure on the probability of reversion is strongest in the biologically relevant case when fitness differences between sensitive and resistant strains in absence of treatment are small.

## Results

### Spread and competition of strains on networks

We investigated the spreading and competition dynamics of sensitive (wild-type) and resistant infectious disease strains using a model of disease spread in a host population with heterogeneous contact structure in presence and absence of treatment (see Methods).

**Fig 1:**
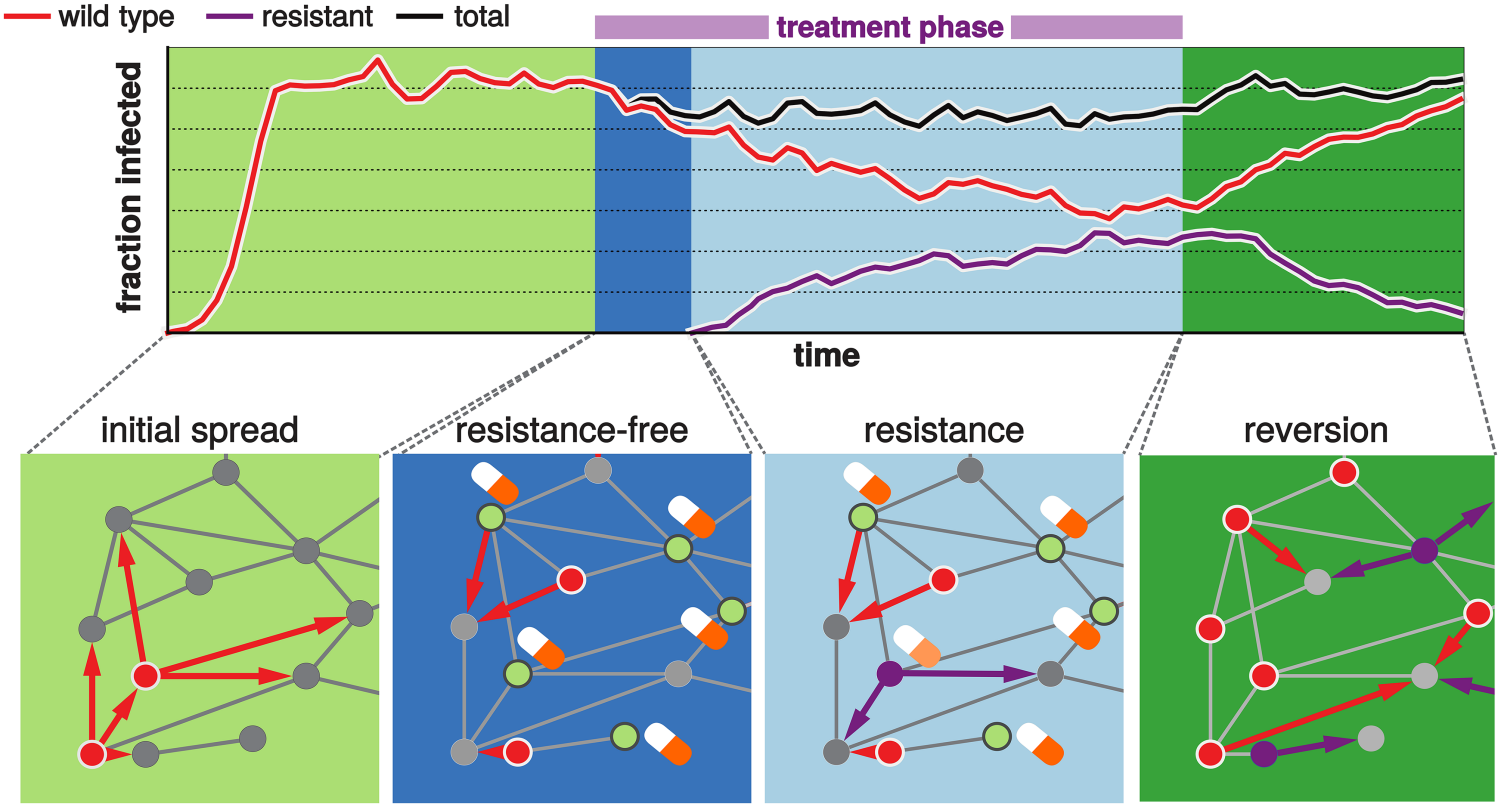
Simulation of the pathogen spread and treatment on a random host network. The different background colors indicate absence (green) and presence (blue) of treatment. The phases in absence of treatment are the initial spread of the wild-type strain (light green) and the potential reversion back to wild type after treatment has stopped (dark green). Treatment is divided into the phases before (dark blue) and after (light blue) the *de novo* emergence of resistance. The solid lines indicate the fraction of hosts infected with either the wild type (red) or the resistant strain (purple). The black line gives the total fraction of infected hosts. The bottom panels illustrate the corresponding phases on the level of the individual hosts connected to each other. During the initial spread there are only two types of hosts: susceptibles (grey) and wild-type infecteds (red). The arrows indicate the transmission events from an infected to a susceptible host. During treatment two new types of hosts arise: In the resistance-free phase we only need to distinguish between treated (green with pill) and untreated (red) infecteds. In the resistance phase we additionally have resistant infected hosts (purple). Finally, in the reversion phase we loose the treated class of hosts and remain with susceptibles, wild-type and resistant infecteds. We generally assume that after recovery individuals are susceptible again and that treatment of resistant infecteds has no effect.

Fig 1 illustrates the distinct phases of the spread and competition of the strains before, during and after treatment.

Initially, the sensitive strain spreads in a fully susceptible host population and reaches an endemic state. Treatment is then initiated on a population level. As a consequence of treatment, new infections with the sensitive strain begin to decline in the host population. Within an individual, however, treatment selects for resistant strains. These can be generated *de novo* within a treated individual and then be transmitted in the host population. Resistant strains are assumed not to be affected by treatment. Hence during treatment the fraction of resistant infections increases in the host population, while the fraction of sensitive infections further declines. Once the fraction of resistant infections has risen to a critical level, treatment is stopped in our simulations. From this point onwards, there is no further generation of *de novo* resistance and the dynamics are governed by the competition of wild-type and resistant strains in absence of treatment. We assume that wild-type and resistant strains can coexist on the level of the host population but not within a single individual. This leads to a competitive exclusion between the strains and therefore competition for the hosts on the between-host level. This assumption completely excludes co-infections and any resulting within-host competition, which allows us to study the impact of the host contact structure in an isolated manner and obtain a clear outcome, i.e. extinction of either of the two strains.

In the long run either reversion to the wild-type or fixation of the resistant strain is observed in the host population. We define the ‘probability of reversion’ back to the wild-type strain, *P*_rev_, as the fraction of simulations in which the resistant strain goes extinct after stopping treatment.

### Probability of reversion to wild type

**Fig 2:**
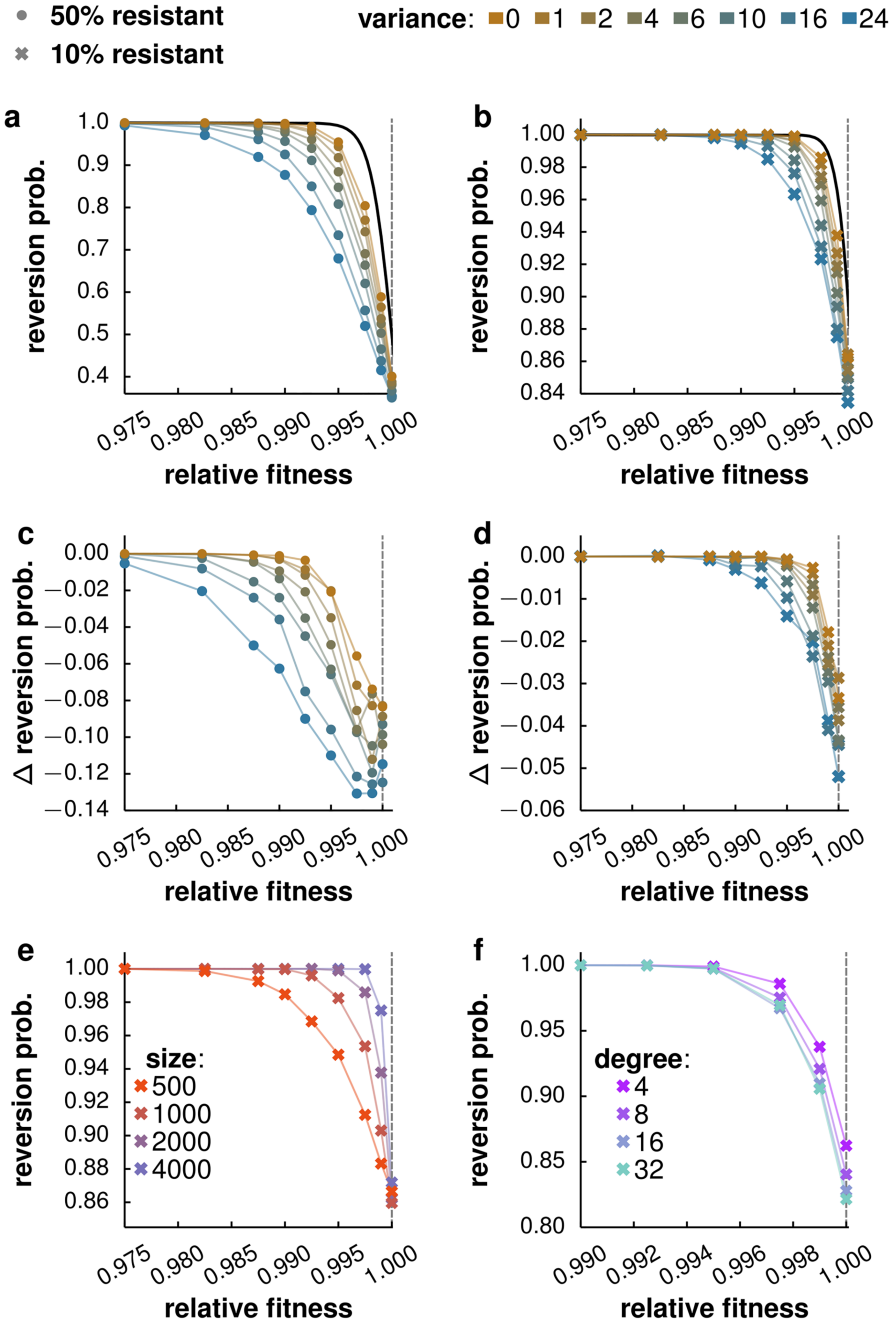
Effect of treatment halt, network size and density on reversion probability. **a,b.** Probability of reversion after a gradual treatment halt as a function of the relative fitness of the resistant strain for critical resistance fractions *f*_*r*_ = 0.5 and 0.1, respectively. Almost certain reversion happens for sufficiently large fitness disadvantages of the resistant strain: relative fitness *s*_*A*_ < 0.975 for a resistant fraction of *f*_*r*_ = 0.5 and *s*_*A*_ < 0.985 for *f*_*r*_ = 0.1. As a reference, the analytical probability, using a Moran model [50], of reversion in a random host population with homogeneous degree is shown (black lines). The model for disease spread (coloured lines) shows generally a smoother transition as compared to the Moran model. In networks with higher variance in degree, the probability of reversion changes more gradually with relative fitness. **c,d.** Difference of the reversion probability between immediate and gradual treatment halt for *f*_*r*_ = 0.5 and 0.1, respectively. In panels **a-d** color gradient indicate increasing variance of the degree distribution of the network. **e.** Probability of reversion as a function of the relative fitness of the resistant strain for host populations with zero degree variance and various system sizes. **f.** Reversion probability as a function of the relative fitness of the resistant strain for host populations with various densities (i.e. mean degrees) and zero variance. The change in connection density is compensated by adapting the transmission rate of the pathogen such that the epidemic threshold is kept constant, *R*_0_ = 3. In all simulations, treatment coverage is complete (*c* = 1) and drug efficacy is half maximal (*e* = 0.5).

The probability of reversion back to the wild type decreases with increasing relative fitness of the resistant strain both for high and low critical fractions of resistance, *f*_*r*_ (Fig 2a and 2b for *f*_*r*_ = 0.5 and *f*_*r*_ = 0.1).

In general, the probability of reversion as a function of the relative fitness has a sigmoidal shape, with an inflection point at a relative fitness of *s*_*A*_ = 1. For low enough relative fitness of the resistant strain (*s*_*A*_ < 0.97), reversion to the wild type happens almost certainly (*P*_rev_ ≈ 1). Equivalently, for high enough relative fitness (*s*_*A*_ > 1.03) the resistant strain almost certainly goes to fixation (*P*_rev_ ≈ 0) (see S1 Fig). The transition from almost certain reversion to the wild type, to almost certain fixation of the resistant strain is less steep for simulated host contact networks than for a fully mixed population and depends on the variance in degree of the network. An increase in variance makes the transition more smooth, i.e. results in a decrease in the probability of reversion for *s*_*A*_ < 1, and an increase in probability of reversion for *s*_*A*_ > 1. As a reference, we report the probability of reversion in the case of a Moran process on a random host population with homogeneous degree, $P_{\text{rev}}^{*}=\frac{1-s_{A}^{-f_{r}N}}{1-s_{A}^{-N}}$ (solid black line in Fig 2a and 2b; ref. [50]). Note, that pathogen competition is not expected to directly match this type of process, as the population of infected hosts is a dynamically changing sub-sample of the entire population: In contrast to the Moran process, transmission does not occur within this sub-sample, but exclusively between the infecteds and the rest of the population. In the limit of large transmission rates, however, the population of infected hosts extends to the entire host population and it can be shown that we recover the Moran process (see S2 Text). In the terminology of a Moran process the previous observation reads: increasing variance in the degree distribution of the host network decreases the effects of selection and hence favours random drift.

The treatment halt on the population level is implemented by not providing treatment for any newly infected individuals, but allowing the individuals currently on treatment to finish their protocol. We refer to this scenario as *gradual treatment halt*. In a more instantaneous scenario, treatment is stopped in all patients at the same time, leading to a discontinuation of the treatment in infected individuals. We refer to this scenario as *immediate treatment halt*. A gradual treatment halt generally decreases the probability of reversion compared to an immediate treatment halt (Fig 2c and 2d). This is expected, since a gradual treatment halt will continue to disfavour the wild-type compared to the resistant strain, because some individuals remain on treatment beyond the end of the treatment phase and therefore have a reduced transmission rate.

The range of relative fitness spanning the transition from almost certain reversion to almost certain extinction narrows with increasing population size (Fig 2e) leading to a step-like transition in the limit of large system size. To assess the impact of connection density we report the probability of reversion for systems with fixed size and zero variance but different mean in the degree distribution (Fig 2f). We compensate for changes in the connection density by adapting the transmission rate such that the epidemic threshold is kept constant, i.e. all systems depict the same basic reproductive ratio of *R*_0_ = 3. Tuning the connection density in this manner has no impact on the reversion probability for relative fitness values that lead to almost certain reversion (*s*_*A*_ < 0.97). For relative fitness values closer to one, an increased density in contacts leads to a reduction of the probability of reversion.

### Effects of network heterogeneity on probability of reversion

**Fig 3:**
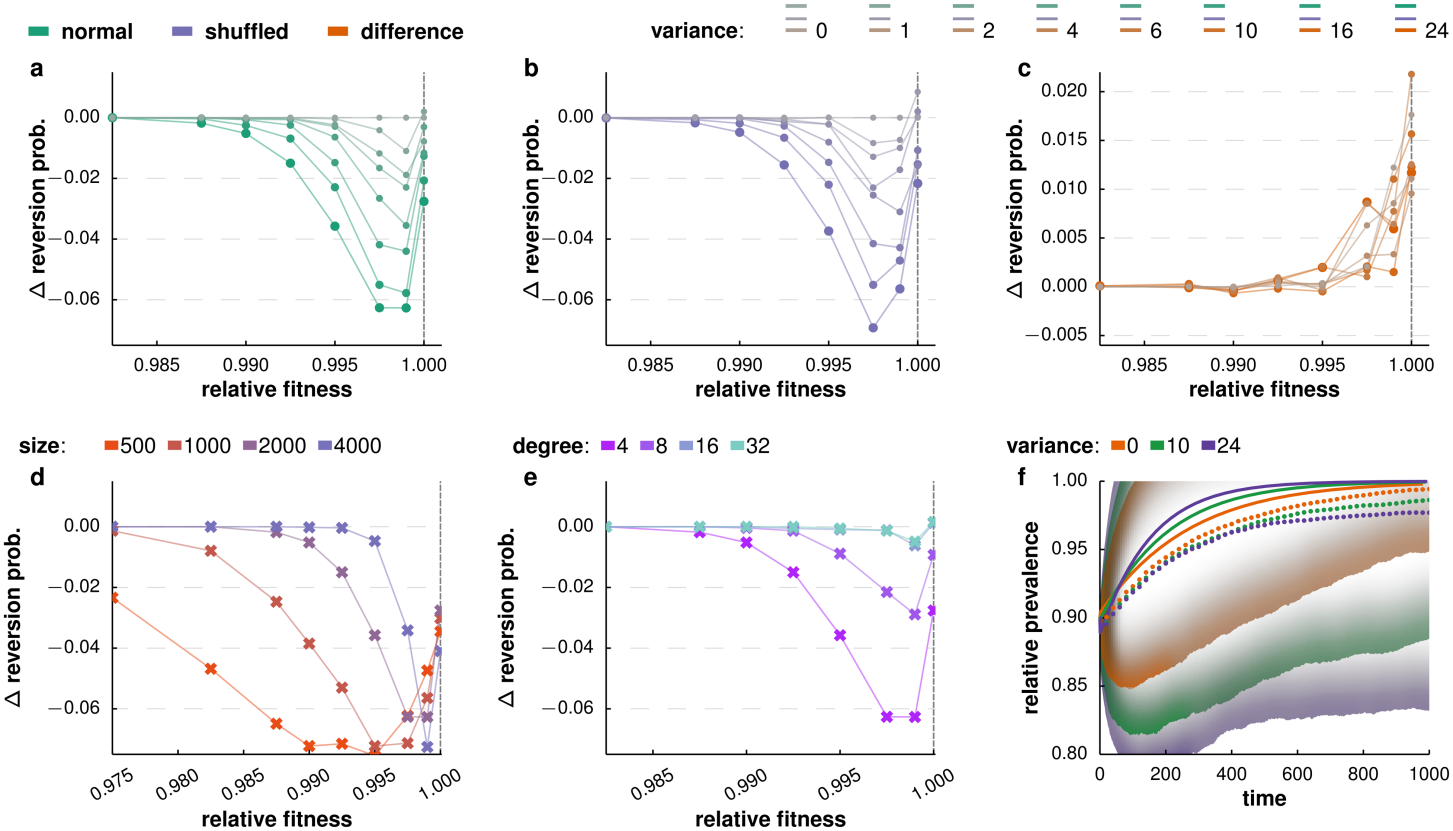
Characterization of the effect of degree variance on reversion probability. **a-c.** The magnitude of the effect of variance in degree of the network relative to zero variance (gray lines) as a function of the relative fitness of the resistant strain, for a fraction of resistant infecteds of *f*_*r*_ = 0.1 at a gradual treatment halt. Panel **a** illustrates the impact of degree variance on the probability of reversion. For values of the relative fitness of the resistant type close to but slightly smaller than 1, an increase in degree variance leads to a substantially lower probability of reversion. Panel **b** illustrates the effect of network occupancy. It reports the magnitude in effect of the variance in degree on the reversion probability in case of a shuffled distribution of the infection type (resistant versus wild type) among all infecteds at the end of treatment. To assess the effect of network occupancy within the infecteds at the end of treatment, panel **c** shows the difference between treatment halt without and treatment halt with shuffling of the infection type. We see that network occupancy has a slightly positive effect on the probability of reversion. **d.** The effect of variance (*σ*^2^ = 24) in degree relative to zero variance as a function of the relative fitness of the resistant strain, for a range of host population sizes. **e.** Impact of host network density on the relative effect of variance (*σ*^2^ = 24) as a function of the relative fitness of the resistant strain. **f.** Relative prevalence of the wild-type strain during the post treatment phase for a relative fitness of the mutant strain of *s*_*A*_ = 0.995. Solid lines show the analytical solution of a two-strain pair approximation. The mean relative prevalence (dotted lines) is lower for network with a higher degree variance. The standard deviation of the mean relative prevalence (outlined with according color gradient) increases with the degree variance, indicating an increase in the magnitude of stochastic noise with increasing variance in degree of the host network.

To further disentangle the effect of network heterogeneity from the effects of relative fitness, we isolated the contribution of variance in degree by comparing the probability of reversion in host networks with non-zero variance to networks with zero variance. Here we consider the case of a gradual treatment halt (see S2 Fig for the scenario with an immediate treatment halt). An increased variance in degree decreases the probability of reversion most strongly for small fitness differences between the wild-type and the resistant strains in absence of treatment (Fig 3a). Because the costs of resistance are generally small for many pathogen-drug combinations, the biologically most relevant parameter range is the range where the effects of contact heterogeneity are expected to be largest. The range of relative fitness values for which variance in degree affects the reversion probability straitens and shifts closer to *s*_*A*_ = 1 with increasing system size (Fig 3d). Increasing the contact density generally reduces the effect of degree variance on the reversion probability (Fig 3e).

Network heterogeneity can influence which strain takes over in two ways: directly, by modulating the competition dynamics in the post-treatment phase; or indirectly, by influencing the positioning of the wild-type and resistant strains as a result of the competition during the treatment phase. To assess the direct impact of variance, we randomized the distribution of wild-type and resistant strains among the infected individuals at the end of the treatment phase and again compared the probability of reversion for networks with non-zero variance to networks with zero variance (Fig 3b). The reduction in probability of reversion persists when randomizing within infected individuals of the networks, indicating that host population structure directly modulates the competition dynamics during the post treatment phase. To assess the indirect impact, we compared the reversion probabilities from these randomised distributions (Fig 3c) to their non-randomised counterparts (Fig 3a). Generally, shuffling the distribution of the resistant and wild-type strains has little effect on the probability of reversion (Fig 3c). Only for excessively small fitness differences (*s*_*A*_ > 0.995) does the distribution of strains among the infected individuals at the end of the treatment phase additionally favour the wild-type strain. We note that this slightly beneficial effect on the reversion probability increases with increasing relative fitness of the resistant strain. While the distribution of resistant and wild-type strains at the end of treatment shows a small effect on the probability of reversion, contact heterogeneity predominantly modulates the competition dynamics directly during the post-treatment phase.

### Modulation of the competition dynamics in the post treatment phase

To get a better understanding of the underlying processes that influence the competition after treatment halt, we investigate the time dynamics of the relative prevalence of the wild-type strain during the post treatment phase. Fig 3f shows mean (dotted lines) and standard deviation (borders of colored areas) of the relative prevalence of the wild-type strain during the post treatment phase after an immediate treatment halt. The simulated mean relative prevalence increasingly deviates from the pair approximation (solid lines) with increasing variance in degree.

If exclusively simulations that revert back to the wild type are considered (see S3 Fig), eventual reversion occurs faster with increasing degree variance. This indicates an increase of reversion rate with increasing degree variance and is consistent with results from an analytical approach using a pairwise approximation model (S4 Fig and S1 Text for further details on the two-strain pairwise approximation approach).

If all simulations are considered, increasing variance leads to slower reversion, inverting the trend observed both in the analytical solution and when considering reverting simulations only. This inversion of the effect of degree variance on the reversion rate can be understood when considering the standard deviation of the relative prevalence (boarder of colored areas in Fig 3f): Increased degree variance leads to an increased standard deviation which, in turn, increases the chance for the wild-type strain to go extinct during the post-treatment phase despite its fitness advantage. As a consequence we observe both lower probability of reversion and a lower mean relative prevalence in networks with high degree variance. Hence, even with an initial abundance of 90% of the wild-type strain, stochastic effects play an important role in the process of reversion. Contact heterogeneity in the host population increases the magnitude of these stochastic effects resulting in a prolonged phase of co-existence of the competing strains.

### Effect of resistant strain positioning at the end of treatment

**Fig 4:**
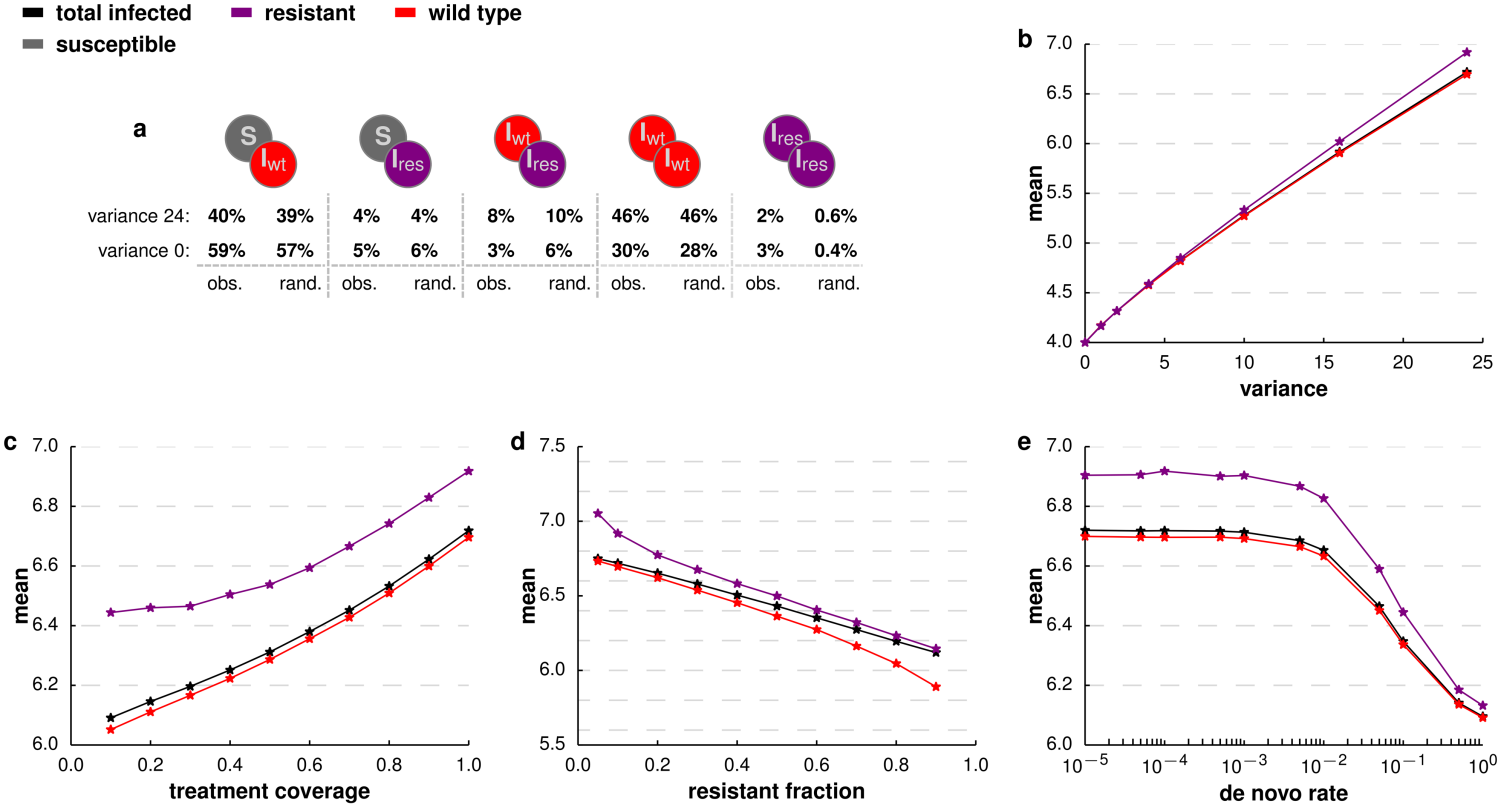
Description of infected population at the end of treatment. **a.** Pairwise contacts at the end of treatment for all possible combinations of pairs involving at least one infected individual. For each infected individual its abundance in a host structures with *σ*^2^ = 24 and zero variance is reported both as observed at the end of treatment (obs.) and after randomisation of the infecteds (rand.). The abundance is given relative to the total number of pairs with at least one infected individual. **b-e.** Average degree of all infected (black), wild-type infected (red), and resistant strain infected (purple) individuals at the end of the treatment phase. Panel **b** shows the mean degree as a function of the degree variance in the network. Increasing variance in the degree distribution of the host network leads to a higher mean degree in the infected individuals. Individuals infected with the resistant strain show an even higher mean degree. Panel **c** illustrates the dependence of the mean degree on the treatment coverage. Panel **d** shows the mean degree as a function of the critical fraction of resistant infected individuals, *f*_*r*_. The advantage of a higher mean degree of the resistant strain vanishes with increasing critical fraction. In panel **e** we show the mean degree as a function of the rate of *de novo* emergence during the treatment phase. The trend of a higher mean degree of infected individuals with an even stronger signal for resistant infecteds is robust throughout a large range of *de novo* emergence rates, breaking down only for unrealistically high rates. If not specified otherwise, all simulations assume a degree variance of *σ*^2^ = 24 of the host network, full treatment coverage, *c* = 1, a drug efficacy of *e* = 0.5, and a *de novo* emergence rate *r* = 0.0001.

To assess the indirect effect of placement of wild-type and resistant strains in the network at the end of the treatment phase, we counted the number of contacts between pairs of individuals of various infection status (Fig 4a).

Randomizing the strains among infected individuals keeps the overall number of susceptible/infected pairs constant, but destroys any non-random pattern of occupancy. Thus, a change in the fraction of a specific type of pair in the non-randomized occupancies could explain the differences in outcome between the randomized and non-randomized cases. The fraction of wild type/wild type and resistant/resistant pairs is higher in the non-randomized occupancies than in the randomized occupancies, while the fraction of wild type/resistant pairs is lower in the non-randomized occupancies (Fig 4a). This indicates a tendency for the resistant strains to aggregate during the treatment phase. Additionally, the fraction of susceptible/resistant pairs is also lower in the non-randomized occupancies (Fig 4a). From the point of view of susceptible individuals, the wild-type strain therefore acts as an insulator from the resistant strain, leading to an advantage of the wild-type strain and thus a higher probability of reversion. During the treatment phase, the wild-type strain is heavily disfavored, i.e. *s*_*A*_ ≫ *s*_*P*_, such that a susceptible individual in contact with a wild-type infected is less likely to contract the diseases than in the case of a susceptible/resistant pair. A higher density of susceptible individuals is thus expected in regions of the host network that are dominated by the wild-type strain. Interestingly, this effect diminishes for networks with higher variance in degree.

For host networks with non-zero variance in degree, a strain gains an advantage from both an increased exposure to susceptible hosts, as well as from occupying individuals with a high degree. Infected individuals generally have a higher degree than randomly chosen individuals in the network (Fig 4b). Additionally, individuals infected with the resistant strain have an even higher degree on average. During the treatment phase, the fitness advantage of the resistant strain thus allows it to occupy nodes with high degree in the network, leading to an advantage independent of the positioning relative to the wild-type infected and susceptible hosts.

The spread of the resistant type over the network during the treatment phase thus generates two effects that influence the reversion. Firstly, there is a difference between resistant and wild-type strains in terms of their likelihood to be connected to a susceptible neighbor and thus their potential to infect a given neighbour. Secondly, the nodes occupied by resistant and wild-type strains differ in their degree and thus the resistant type has more neighbours to spread to. The first effect favours the wild type, while the second effect favours the resistant during reversion. With increasing variance in contact structure the first effect is weakened (Fig 4a) and the second strengthened (Fig 4b), resulting in an overall lower probability of reversion for high variance.

The difference in mean degree between wild-type and resistant infecteds is consistent over a vast range of treatment coverage (Fig 4c) and robust with regard to the fraction of resistants at treatment halt (Fig 4d). Also, the effect persists across a wide range of *de novo* rates of resistance, and breaks down only at rates that are unrealistically high.

## Discussion

Our simulations reveal the following key results: i. Increasing variance in contact structure lowers the probability of reversion. ii. Stochastic effects dominate the competition phase after treatment halt even when both strains represent a substantial fraction of the population. iii. The distribution of infected individuals at the end of the treatment phase is highly specific and influences the reversion dynamics.

Given the inherent simplifications of a random network with a heterogeneous distribution of contacts, we caution against taking our quantitative results as representative for the magnitude of effects to be expected in real contact networks. We expect, however, that in real systems, qualitatively the mentioned effects to be present.

From an evolutionary perspective, i. suggests that treatment in a homogeneous host contact structure would lead to a stronger selection of resistant strains with increased transmissibility as compared to treatment in a more heterogeneous host population. It has been repeatedly hypothesised that an increase in virulence correlates with an increase in transmissibilty [51–54]. Under this assumption, our findings extend conclusions from earlier studies suggesting that more homogeneous contact patterns [46] and increased global connectivity in spatially structured populations [36–38] enhance the evolution of virulence: homogeneity in the host population structure fosters the co-selection of resistance and virulence under treatment.

What is responsible for the effect of variance on reversion probability? Previously it has been shown that the fitter mutant has a disadvantage when invading from a single individual into a resident population of the wild type [47]. This effect may, at least in part, be due to stochastic effects in small populations [55]. In our simulations, however, the fitter variant (i.e. the wild type) is present at high frequencies of 50% or even 90% where stochastic effects due to small population size are negligible. Another potential explanation would be that the absolute prevalence at treatment stop affects the probability of reversion and that variance in the host degree distribution merely acts on the absolute prevalence. We tested this possibility but found no evidence that changes in the order of 5% to 10% in total prevalence at treatment halt had any effect on the probability of reversion (see S3 Text). The randomizations of the network occupancy at the end of the treatment phase show that contact heterogeneity in the host structure does not only impact the configuration of resistant and wild-type infecteds at the end of treatment, but also shapes the competition dynamics during the post treatment phase (Fig 3). In fact, for a large range of frequencies of the fitter strain, most of the observed effect of variance in degree on the probability of reversion is due to the increase in stochastic effects during this competition phase. Recent works [56, 57] have addressed the prolonged co-existence of wild-type and resistant strains observed in many real-world diseases. The shift towards random drift, i.e. mitigation of selection pressures resulting from fitness differences when degree heterogeneity increases, is an additional factor favoring a prolonged co-existence. It is important to note that the stochastic nature of the reversion process does not primarily result from effects of small population size, but rather the small fitness differences between the strains, a property that is found in real systems. Thus ii. is in contrast to what has been reported in the case of self-limiting dynamics, where the outcome of the simultaneous spread of two pathogens is largely determined by the initial proportions of the pathogens [44]. This is also reflected by the decent quality of predictions from analytical models [46, 58] for self-limiting dynamics.

The specific distribution of infecteds at the end of treatment, i.e. result iii., can be characterized by two distinct features in the occupancy patterns, both affecting reversion (Fig 4): First, we observe that individuals infected with the resistant strain tend to be aggregated at the end of the treatment phase. This implies that at the beginning of the reversion phase the resistant infected individuals have a lower per contact probability to be connected to a susceptible individual thus favouring reversion to wild type. This observation is surprising, given that the underlying model for the contact structure is a random graph providing no topological support for aggregation. We thus hypothesize this aggregation tendency to potentially have a much bigger impact on structures that provide non-random topologies.

Second, it has previously been shown that the sub-population of infected individuals tends to have a higher average degree [32]. We find that that the same holds true for the resistant sub-population among the infecteds: At the end of treatment, individuals infected with the resistant strain tend to have a higher mean number of contacts than those infected with the wild-type strain. The higher number of contacts leads to an increased chance of transmission for the resistant strain and thus lowers the probability of reversion.

The first effect promotes reversion while the second effect obstructs it. However, both share a common tendency with increasing degree variance in the networks: (a) the tendency of resistant strain to aggregate decreases, reducing the positive effect on the probability of reversion and (b) the difference in mean contact number of resistant and wild-type infecteds increases, further favoring the resistant strain.

Combining the above described effects leads to two main conclusions: First, an increase in heterogeneity in the host network diminishes the probability of reversion back to the wild type. Second, the impact of heterogeneity is most pronounced for small fitness differences between the resistant and the wild-type strain in the absence of treatment.

Our findings consider a competition process of two strains under a SIS-dynamics with a first-come-first-serve exclusion on the level of a single host. It goes without saying, that the observed competition and its outcome are consequences of this particular model choice. The SIS-type model—the simplest mathematical model of an endemic disease [68]—allows to study the effect of host heterogeneity on a continual pathogen evolution. It is, however, not suitable to investigate the case of successive single-wave outbreaks in a population that is partially immune. The effect of heterogeneity in the host structure on pathogen evolution in the case of single-wave dynamics has been covered by several studies [43–46, 49]. They make use of SIR-type models which are more suitable for this type of outbreaks. SIR-type models would require additional modeling of demographic changes in the host population for an endemic state to be possible.

The first-come-first-serve exclusion is an extreme case of interaction amongst the strains as it excludes both the possibility of a simultaneous infection of a single host by both strains (co-infection) and a displacement of one strain by the other in a currently infected host (super-infection). Conversely, a complete absence of interaction will lead to simultaneous but independent epidemics of both strains. In-between those two extremes, strain interactions can occur in a multitude of forms and will depend on the particular real world disease.

The low fitness cost of resistance mutations is likely one of the key factors contributing to the observation that reversion to wild type is often slow or even absent. Interestingly, the network effects we observe here are most pronounced exactly at these small fitness differences. The reason why fitness costs are often small is that the potentially larger direct costs of resistance conferring mutations are often alleviated by compensatory mutations [20–22]. Reversion from a genotype that carries both resistance and compensatory mutations may require crossing a fitness valley, which obstructs the reemergence of sensitivity, as has been shown in *in vitro* studies [23]. This argument, however, only applies when the wild-type strain has become extinct. While extinction of the wild type might occur in individual patients, it is unlikely to occur on the epidemiological level. As soon as there is even a small fraction of wild type in the population, the outcome of the dynamics is a matter of competition, and does not require *de novo* reemergence of the sensitive wild type. Although the mechanism is entirely different, treatment on the population level with heterogeneous host contact patterns leads to a similar phenomenon: the path to resistance is easier than the way back. The network structure of the host population obstructs the reversion back to the wild type. The small fitness difference between resistant and sensitive strains in absence of treatment not only result in a low reversion rate, but, together with heterogeneity in contact number, the small fitness difference additionally results in a reduced probability of reversion.

Individual variations in contribution to disease transmission were shown to be present across the bard of infectious diseases [59, 60]. This is particularly important for sexually transmitted diseases where individual variations are thought to occur through differences in patterns of sexual-partner acquisition [61–65] thus leading to heterogeneous contact structures.

Given this generally heterogeneous nature of host transmission patterns and the maximal impact of the here described network effects in biologically relevant fitness differences, we expect our findings to be of general relevance. It would be interesting to test on the basis of simulations on realistic real world networks the strength of the here described effects. The other option is to explore by simulations what type of networks would show these effects most strongly. Both directions we decided, for reasons of scope, to leave for further studies.

## Materials and methods

### Simulating SIS dynamics on heterogeneous contact networks

We modelled heterogeneous contact structure in the population using random networks with degrees distributed according to a discretized gamma distribution. This allows us to keep the mean fixed but tune the variance in number of contacts per individual. For a given mean *μ* and variance *σ*^2^ the scale *θ* and shape *k* parameter of the gamma distribution is given by

$$\begin{array}{ccc} \theta & = & \frac{\sigma^{2}}{\mu } \end{array}$$ (1)

$$\begin{array}{ccc} k & = & \frac{\mu^{2}}{\sigma^{2}} \end{array}$$ (2)

From the generated distribution we then draw for each node in the network a value and round it to define its degree. We then use a stub connecting algorithm to generate a contact network [66].

The SIS dynamics on the network are simulated using the Gillespie next reaction method [67]. In brief, starting from the first infected individual in the network we draw the duration of its infection from an exponential distribution with recovery rate parameter *γ* and the times to infection of all its neighbors from an exponential distribution with transmission rate parameter *β*. Then, we record the time of recovery as well as those time points of infection that occurred prior to recovery of the individual in a queue of events. The algorithm then proceeds to the next event in the queue. In case this event is an infection and the node to infect is susceptible, the above procedure is repeated. Note, that the condition to infect only susceptible nodes implies that there is no super-infection. In case the event is a recovery, the status of the infected node is reset to susceptible.

### Implementing treatment

During treatment, infected individuals either do or do not receive treatment with a probability *c*, the treatment coverage. Treatment has two consequences: Firstly, during the time period in which an individual receives treatment, the infection rate is reduced by a factor 1 − *e*, where *e* is the efficacy of treatment in preventing transmission. Secondly, treatment can change a wild-type infection into a resistant one. A time point for such an event is determined according to an exponential distribution with a rate of *de novo* emergence *r*. In case this event occurs prior to recovery, it replaces the recovery event in the queue, the status of the node is changed from wild-type to resistant, and a new recovery event for this node is generated according to the parameters of the resistant strain. All transmission events after the *de novo* emergence are discarded and replaced by new transmission events generated according to the transmission rate of the resistant strain *β*_*res*_.

The treatment phase ends when the fraction of resistant infections among the infecteds reaches a value *f*_r_. We implement the end of treatment in the population considering two scenarios: In the gradual treatment halt scenario, patients who are already on treatment continue therapy, but no newly infected individuals receive treatment. In the immediate treatment halt scenario, treatment is stopped in all individuals simultaneously. The immediate treatment halt necessitates that all transmission events are discarded at the start of treatment and are re-generated with updated transmission parameters. The gradual treatment halt implies that the transmission rate parameter is altered in newly infecteds only.

### Simulation of the different phases

The simulations proceed though 3 distinct phases: The initial phase, the treatment phase and the post treatment phase. At the end of each phase the host contact networks, along with its epidemic state is saved.

In the initial phase, we first infect a randomly chosen individual in the population with the wild-type strain. This individual then infects neighbors at a rate *β*_wt_ over the duration of its infection, and recovers at rate *γ*. The initial spreading phase ends when the frequency of wild-type infected individuals reaches a quasi-steady state after a sufficiently long burn-in phase. In case the infection dies out before quasi steady state, the simulation is restarted. Once the quasi steady state is reached, we halt the simulation and store the network and its epidemic state. Subsequently, the treatment phase starts. Wild-type infected individuals that receive treatment infect at a rate *β*_wt_(1 − *e*), individuals infected with the resistant strain transmit at a rate *β*_res_, and recover with the same rate, *γ*, as wild-type infecteds. We assume that the cost of resistance is small relative to the effect of treatment on the wild-type strain and thus approximate the average relative fitness of resistant versus wild-type strains during the treatment phase as a function of the treatment only: *s*_*P*_ = *β*_*res*_/(*β*_wt_(*c*(1 − *e*) + (1 − *c*))) ≈ 1/(1 − *ce*). After treatment is stopped, the post treatment phase starts. In this phase the simulations continue until either of the two strains completely disappears from the population. Here the fitness of the resistant relative to the wild-type strain is simply given by the ratio of their transmission rates, *s*_*A*_ = *β*_res_/*β*_wt_.

### Simulation parameters

Unless otherwise specified, we used a network size of *N* = 2000 and a mean degree of *μ* = 4 at varying levels of variance, *σ*^2^ = 0, 1, 2, 4, 6, 10, 16, 24. For each variance level we generate 1000 networks. The transmission and recovery rate are chosen such that the resulting basic reproductive ratio in the case of zero variance is: $R_{0}=\left(\mu +\frac{\sigma^{2}}{\mu }-1\right)\beta_{\text{wt}}/\gamma =3$ [68]. The initial phase is run twice on each generated network. The treatment phase is then run three independent times for all saved states of the initial phase and for all sets of treatment parameter. We choose treatment efficacy, *e* = 0.5, and complete treatment coverage, *c* = 1, throughout. The *de novo* emergence rate is, if not specified otherwise, *r* = 0.0001. The critical levels at which treatment is halted are *f*_r_ = 0.1 and 0.5. The post treatment phase is then run on each output of the treatment phase and for each transmission rate of the resistant strain. In this manner we end up with at least 5000 simulations of the reversion dynamics, this for each parameter combination.

For the resistant strain a set of transmission rates is chosen such that the relative fitness of the resistant strain *S*_*A*_ = *β*_res_/*β*_wt_ ranges from 0.975 to 1.025.

### Shuffling

To test the effect of network occupancy on the probability of reversion to wild type we shuffled the status of wild-type versus resistant infected individuals at the end of the treatment phase, therefore keeping *f*_*r*_ constant. When shuffling at the end of the treatment phase, we first discard all future infection events. Then we shuffle the status within all infecteds, with the status of an individual being a pathogen strain and a time to recover. Finally, we redraw infection events for all neighbours of the infected individuals.

### Software

EndemicPy, the software package used in this study is freely available on GitHub [69].

## Supporting information

S1 Fig Effect of gradual and immediate treatment halt on reversion probability. Probability of reversion after treatment halt as a function of the relative fitness of the resistant strain for critical resistance fractions *f*_*r*_ = 0.5 (**a** and **c**) and 0.1 (**b** and **d**). Almost certain reversion or fixation happens for sufficiently large fitness differences: relative fitness *s*_*A*_ < 0.975 or *s*_*A*_ > 1.025 for a resistant fraction of *f*_*r*_ = 0.5 and *s*_*A*_ < 0.95 or *s*_*A*_ > 1.1 (limit not visible) for *f*_*r*_ = 0.1. As a reference, the analytical probability of reversion in a death-birth process is shown (black lines). The model for disease spread (coloured lines) shows generally a smoother transition as compared to the death-birth process. In networks with higher variance in degree, the probability of reversion changes more gradually with relative fitness. **a-b.** show the probability of reversion after a gradual treatment halt and **c-d.** after an immediate treatment halt. Color gradient indicate increasing variance of the degree distribution of the network. In all simulations, treatment coverage is complete (*c* = 1) and drug efficacy is half maximal (*e* = 0.5).
(PDF)

S2 Fig Effect of network occupancy, size and density on reversion probability. **a-c.** The magnitude of the effect of variance in degree of the network relative to zero variance (gray lines) as a function of the relative fitness of the resistant strain, for a fraction of resistant infecteds of *f*_*r*_ = 0.1 at a immediate treatment halt. Panel **a** illustrates the impact of degree variance on the probability of reversion. For values of the relative fitness of the resistant type close to but slightly smaller than 1, an increase in degree variance leads to a substantially lower probability of reversion. Panel **b** illustrates the effect of network occupancy. It reports the magnitude in effect of the variance in degree on the reversion probability in case of a shuffled distribution of the infection type (resistant versus wild type) among all infecteds at the end of treatment. To assess the effect of network occupancy within the infecteds at the end of treatment, panel **c** shows the difference between treatment halt without and treatment halt with shuffling of the infection type. We see that network occupancy has a slightly positive effect on the probability of reversion.
(PDF)

S3 Fig Relative prevalence of the wild-type strain during the post treatment phase. Colored lines show the analytical solution of a two-strain pair approximation. Triangle markers indicate the mean relative prevalence of runs conditioned on reverting back to the wild type (green and orange triangles overlap). The mean relative prevalence without a condition on the outcome (circle markers) is lower for network with a higher degree variance. The standard deviation of the mean relative prevalence (outlined with according color gradient) without condition on the outcome increases with the degree variance, indicating an increase in the magnitude of stochastic noise with increasing variance in degree of the host network.
(PDF)

S4 Fig Extinction rate of the less fit strain. Left panel: Calculated decay rate (lines) and numerical approximations of the exponential decay (points) for different fitness differences. Right panel: Extinction rate as a function of the fitness difference for various degree variances. See S1 Text for further details on the two-strain pair approximation approach.
(PDF)

S1 Text Pair approximation for two strains on a graph. (PDF)

S2 Text Limit case death-birth process. (PDF)

S3 Text Impact of absolute prevalence at treatment stop. (PDF)
