# Supplementary figures and images for: Host population structure impedes reversion to drug sensitivity after discontinuation of treatment
Source: PLoS Comput Biol. 2017 Aug 21;13(8):e1005704. doi: 10.1371/journal.pcbi.1005704 (PMC5602665; doi:10.1371/journal.pcbi.1005704)

● 50% resistant

\* 10% resistant

variance: 0 1 2 4 6 10 16 24

gradual

immediate

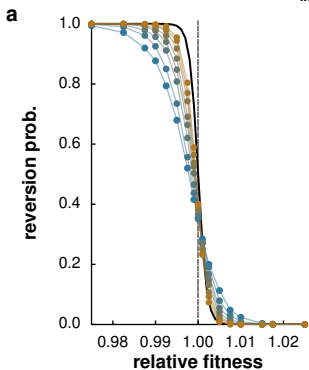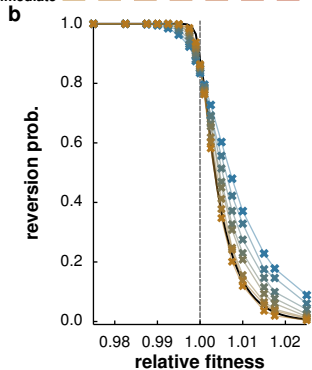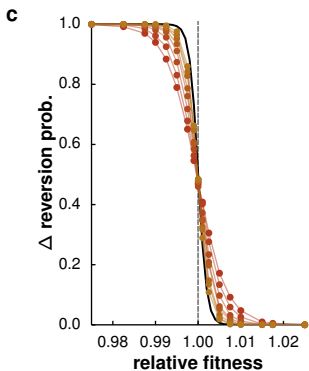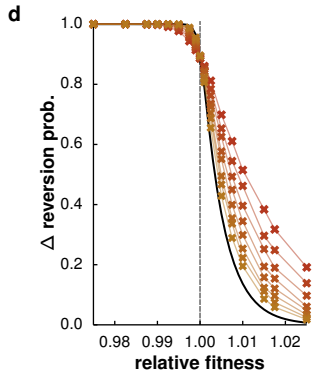

Supplement: S1 Fig — Probability of reversion after treatment halt as a function of the relative fitness of the resistant strain for critical resistance fractions fr = 0.5 (a and c) and 0.1 (b and d). Almost certain reversion or fixation happens for sufficiently large fitness differences: relative fitness sA < 0.975 or sA > 1.025 for a resistant fraction of fr = 0.5 and sA < 0.95 or sA > 1.1 (limit not visible) for fr = 0.1. As a reference, the analytical probability of reversion in a death-birth process is shown (black lines). The model for disease spread (coloured lines) shows generally a smoother transition as compared to the death-birth process. In networks with higher variance in degree, the probability of reversion changes more gradually with relative fitness. a-b. show the probability of reversion after a gradual treatment halt and c-d. after an immediate treatment halt. Color gradient indicate increasing variance of the degree distribution of the network. In all simulations, treatment coverage is complete (c = 1) and drug efficacy is half maximal (e = 0.5). (PDF) [file pcbi.1005704.s001.pdf]

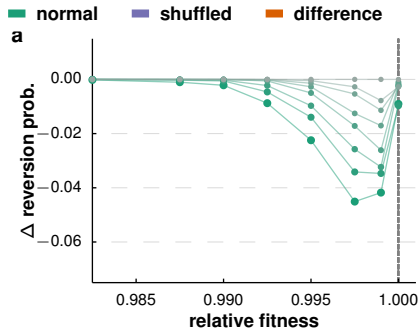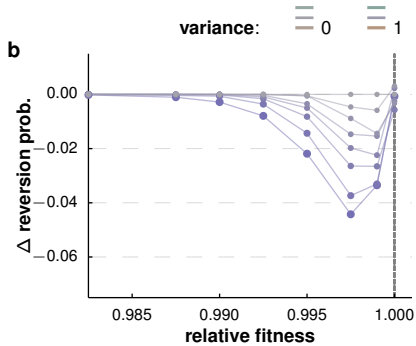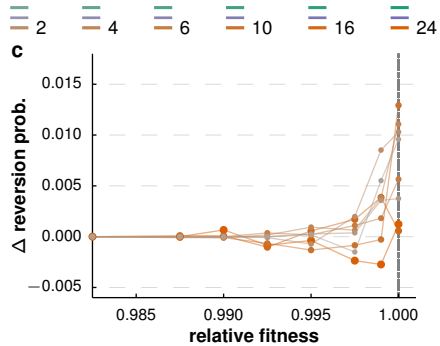

Supplement: S2 Fig — a-c. The magnitude of the effect of variance in degree of the network relative to zero variance (gray lines) as a function of the relative fitness of the resistant strain, for a fraction of resistant infecteds of fr = 0.1 at a immediate treatment halt. Panel a illustrates the impact of degree variance on the probability of reversion. For values of the relative fitness of the resistant type close to but slightly smaller than 1, an increase in degree variance leads to a substantially lower probability of reversion. Panel b illustrates the effect of network occupancy. It reports the magnitude in effect of the variance in degree on the reversion probability in case of a shuffled distribution of the infection type (resistant versus wild type) among all infecteds at the end of treatment. To assess the effect of network occupancy within the infecteds at the end of treatment, panel c shows the difference between treatment halt without and treatment halt with shuffling of the infection type. We see that network occupancy has a slightly positive effect on the probability of reversion. (PDF) [file pcbi.1005704.s002.pdf]

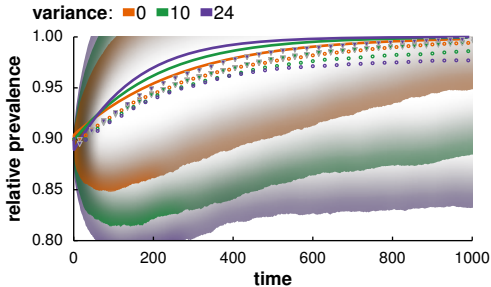

Supplement: S3 Fig — Colored lines show the analytical solution of a two-strain pair approximation. Triangle markers indicate the mean relative prevalence of runs conditioned on reverting back to the wild type (green and orange triangles overlap). The mean relative prevalence without a condition on the outcome (circle markers) is lower for network with a higher degree variance. The standard deviation of the mean relative prevalence (outlined with according color gradient) without condition on the outcome increases with the degree variance, indicating an increase in the magnitude of stochastic noise with increasing variance in degree of the host network. (PDF) [file pcbi.1005704.s003.pdf]

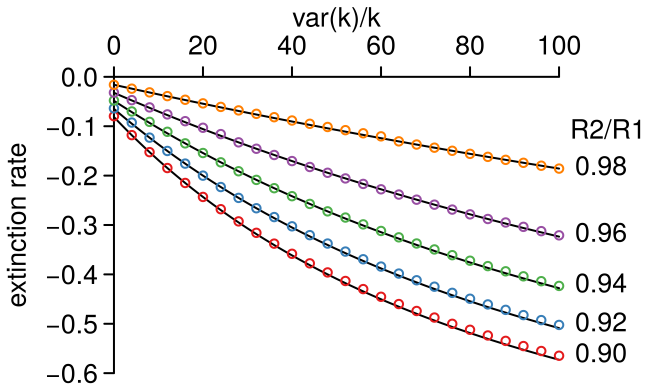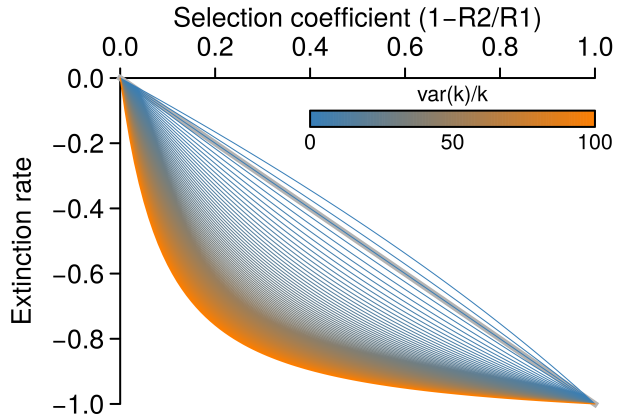

Supplement: S4 Fig — Left panel: Calculated decay rate (lines) and numerical approximations of the exponential decay (points) for different fitness differences. Right panel: Extinction rate as a function of the fitness difference for various degree variances. See S1 Text for further details on the two-strain pair approximation approach. (PDF) [file pcbi.1005704.s004.pdf]
