# Supplementary material for: Host population structure impedes reversion to drug sensitivity after discontinuation of treatment
Source: PLoS Comput Biol. 2017 Aug 21;13(8):e1005704. doi: 10.1371/journal.pcbi.1005704 (PMC5602665; doi:10.1371/journal.pcbi.1005704)
Supplement: S1 Text — (PDF) [file pcbi.1005704.s005.pdf]

Host Population Structure Impedes Reversion to Drug Sensitivity After Discontinuation of Treatment:

Supporting Information

Jonas I Liechti[1,*], Gabriel E. Leventhal[1,2], Sebastian Bonhoeffer[1],

1 Institute for Integrative Biology, ETH Zürich, 8092 Zürich, Switzerland
2 Department of Civil and Environmental Engineering, Massachusetts Institute of Technology (MIT), Cambridge, MA, 02139, U.S.A.

* jonas.liechti@env.ethz.ch

Pair approximation for two strains on a graph

We consider the SIS spread of two pathogen strains on a random graph. The strains have transmission rates β_1 and β_2, and recovery rates γ_1 and γ_2, respectively. Individuals can only be infected by one disease at a time. Expressing the SIS dynamics in terms of pairs [1] we have:

$$\frac{d[S]}{dt} = -\beta_1[SI_1] - \beta_2[SI_2] + \gamma_1[I_1] + \gamma_2[I_2],$$
$$\frac{d[I_1]}{dt} = \beta_1[SI_1] - \gamma_1[I_1],$$
$$\frac{d[I_2]}{dt} = \beta_2[SI_2] - \gamma_2[I_2].$$

The differential equation for $[SI_1]$ pairs is:

$$\frac{d[SI_1]}{dt} = -\beta_1[SI_1] - \gamma_1[SI_1] - \beta_1[I_1SI_1] - \beta_2[I_2SI_1] + \gamma_1[I_1I_1] + \gamma_2[I_2I_1] + \beta_1[SSI_1]. \quad (1)$$

This equation reflects the fact that $[SI_1]$ pairs can be produced or lost through the following processes (in order of the terms appearing in the above equation): infection of the S individual in an $[SI_1]$ pair; recovery of the I individual in an $[SI_1]$ pair; infection of the S individual in $[SI_1]$ pair from a third party I_1 individual; ditto for I_2; recovery of an I_1 individual in an $[I_1I_1]$ pair; recovery of an I_2 individual in an $[I_2I_1]$ pair; and infection of an $[SS]$ pair by an third party I_1 individual. Analogously, the equation for $[SI_2]$ is:

$$\frac{d[SI_2]}{dt} = -\beta_2[SI_2] - \gamma_2[SI_2] - \beta_1[I_2SI_1] - \beta_2[I_2SI_2] + \gamma_1[I_1I_2] + \gamma_2[I_2I_2] + \beta_2[SSI_2]. \quad (2)$$

Using the population size N we can express the number of susceptibles as

$$[S] = N - ([I_1] + [I_2]). \quad (3)$$

The number of $[SS]$, $[I_1I_1]$ and $[I_2I_2]$ pairs can be expressed using the number of connections k:

$$[SS] = k[S] - [SI_1] - [SI_2], \quad (4a)$$
$$[I_1I_1] = k[I_1] - ([SI_1] + [I_2I_1]), \quad (4b)$$
$$[I_2I_2] = k[I_2] - ([SI_2] + [I_1I_2]). \quad (4c)$$

We can express the coupling term $[I_1I_2]$ as,

$$\frac{d[I_1I_2]}{dt} = -(\gamma_1 + \gamma_2)[I_1I_2] + (\beta_1 + \beta_2)\frac{k_1}{k}\frac{[SI_1][SI_2]}{[S]}, \quad (5)$$

where k_1 is the excess degree relating to the degree distribution by $k_1 = var(k)/k + k - 1$ (see [2]). Further we used the triplet closure:

$$[XYZ] = \frac{k_1}{k}\frac{[XY][YZ]}{[Y]}.$$

Using equations 3, 4a, 4b, 4c and 5 along with the triplet closure we rewrite equations 1 and 2 and get the ordinary differential equations that govern the two-strain pair approximation:

$$\begin{aligned}
\frac{d[I_1]}{dt} &= \beta_1[SI_1] - \gamma_1[I_1] \\
\frac{d[I_2]}{dt} &= \beta_2[SI_2] - \gamma_2[I_2] \\
\frac{d[SI_1]}{dt} &= \left(\beta_1(k_1-1) - 2\gamma_1\right)[SI_1] + \gamma_1 k[I_1] + (\gamma_2 - \gamma_1)[I_1I_2] - \frac{k_1}{k}\frac{2\beta_1[SI_1]^2}{N - ([I_1]+[I_2])} - \frac{k_1}{k}\frac{(\beta_1+\beta_2)[SI_1][SI_2]}{N - ([I_1]+[I_2])} \\
\frac{d[SI_2]}{dt} &= \left(\beta_2(k_1-1) - 2\gamma_2\right)[SI_2] + \gamma_2 k[I_2] + (\gamma_1 - \gamma_2)[I_1I_2] - \frac{k_1}{k}\frac{2\beta_2[SI_2]^2}{N - ([I_1]+[I_2])} - \frac{k_1}{k}\frac{(\beta_1+\beta_2)[SI_1][SI_2]}{N - ([I_1]+[I_2])} \\
\frac{d[I_1I_2]}{dt} &= -(\gamma_1+\gamma_2)[I_1I_2] + \frac{k_1}{k}\frac{(\beta_1+\beta_2)[SI_1][SI_2]}{N - ([I_1]+[I_2])}
\end{aligned}$$

We non-dimensionalize by dividing by the population size, $x_1 = [I_1]/N$ and $x_2 = [I_2]/N$, and the total number of pairs, $y_1 = [SI_1]/(kN)$, $y_2 = [SI_2]/(kN)$ and $y_3 = [I_1I_2]/(kN)$.

$$\begin{aligned}
\frac{dx_1}{dt} &= \beta_1 k y_1 - \gamma_1 x_1 \\
\frac{dx_2}{dt} &= \beta_2 k y_2 - \gamma_2 x_2 \\
\frac{dy_1}{dt} &= \left(\beta_1(k_1-1) - 2\gamma_1\right) y_1 + \gamma_1 x_1 + (\gamma_2 - \gamma_1) y_3 - k_1 \frac{2\beta_1 y_1^2 + (\beta_1+\beta_2) y_1 y_2}{1 - (x_1+x_2)} \\
\frac{dy_2}{dt} &= \left(\beta_2(k_1-1) - 2\gamma_2\right) y_2 + \gamma_2 x_2 - (\gamma_2 - \gamma_1) y_3 - k_1 \frac{2\beta_2 y_2^2 + (\beta_1+\beta_2) y_1 y_2}{1 - (x_1+x_2)} \\
\frac{dy_3}{dt} &= -(\gamma_1+\gamma_2) y_3 + k_1 \frac{(\beta_1+\beta_2) y_1 y_2}{1 - (x_1+x_2)}
\end{aligned}$$

Furthermore, if we rescale time such that $a = \gamma_1 t$, and define $R_j = \beta_j/\gamma_j$ and $c = \gamma_2/\gamma_1$:

$$\begin{aligned}
\frac{dx_1}{da} &= kR_1 y_1 - x_1 \\
\frac{dx_2}{da} &= c\left(kR_2 y_2 - x_2\right) \\
\frac{dy_1}{da} &= \left(R_1(k_1-1) - 2\right) y_1 + x_1 + (c-1) y_3 - k_1 \frac{2R_1 y_1^2 + (R_1 + cR_2) y_1 y_2}{1 - (x_1+x_2)} \\
\frac{dy_2}{da} &= c\left(R_2(k_1-1) - 2\right) y_2 + c x_2 - (c-1) y_3 - k_1 \frac{2cR_2 y_2^2 + (R_1 + cR_2) y_1 y_2}{1 - (x_1+x_2)} \\
\frac{dy_3}{da} &= -(1+c) y_3 + k_1 \frac{R_1 + cR_2}{1 - (x_1+x_2)} y_1 y_2
\end{aligned}$$

Equilibrium solution for a single strain. For a single strain, $x_2 = y_2 = y_3 = 0$, and the non-trivial equilibrium solution is,

$$x_1^* = \frac{k(R_1(k_1+k-1) - 2)}{2(k_1-k) + R_1 k(k_1+k-1)}, \tag{6a}$$

$$y_1^* = \frac{R_1(k_1+k-1) - 2}{R_1(2(k_1-k) + R_1 k(k_1+k-1))}. \tag{6b}$$

Simultaneous spread. We first consider the simultaneous spread of the two strains in a completely susceptible population. Each strain starts from a single infected individual, $x_1(0) = x_2(0) = 1/N$. These individuals are considered to be "far apart" in the network, such that $y_1(0) = y_2(0) = 1/N$ and $y_3 = 0$. An example of spreading dynamics is shown in Fig. S1. The dynamics are split into two phases: In a first phase, the two strains are rather unaffected by one another. In a second phase, once an important fraction of the population is infected, competition for susceptibles between the two strains takes over. Because the second strain spreads slower ($R_2 < R_1$), it is eventually outcompeted by the first strain.

Note, that for relatively small differences in R the competition phase is much longer than the initial spreading phase. A closer look at the two phases reveals that the initial spread is characterized by an exponential increase in the prevalence of strain 2, followed by an exponential decrease of the prevalence of strain 2 during the competition phase (Fig. S2). Evidently, the longer competition phase is reflected in a slower rate of decline.

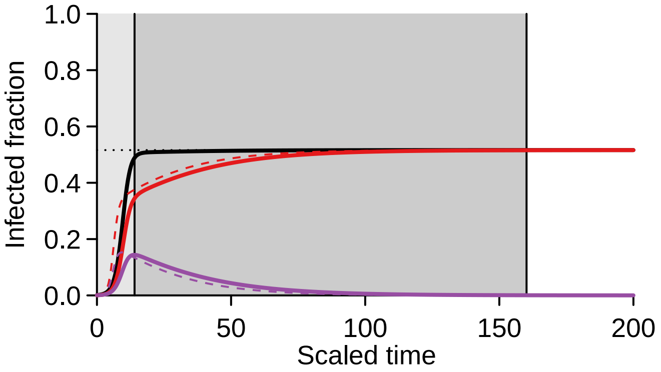

Figure S1: **Concurrent spread of two strains on a random graph.** Solid lines show the dynamics on a random graph, dashed lines show the dynamics for a mean field model (and Poisson). Note, that the R_1 in the mean field model is adjusted, such that the equilibrium prevalence for a single strain is the same. Parameters: $k = 4, R_1 = 0.6, R_2/R_1 = 0.95$.

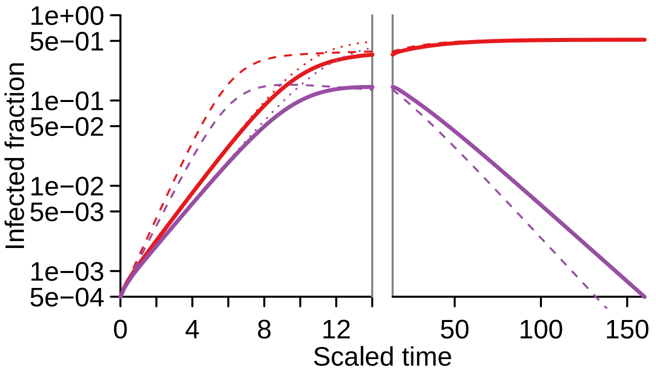

Figure S2: **Closer look at the two phases of initial spread followed by competition.** Solid lines show the dynamics on a random graph, dashed lines for the mean-field case. The exponential decay in the competition phase is clearly visible.

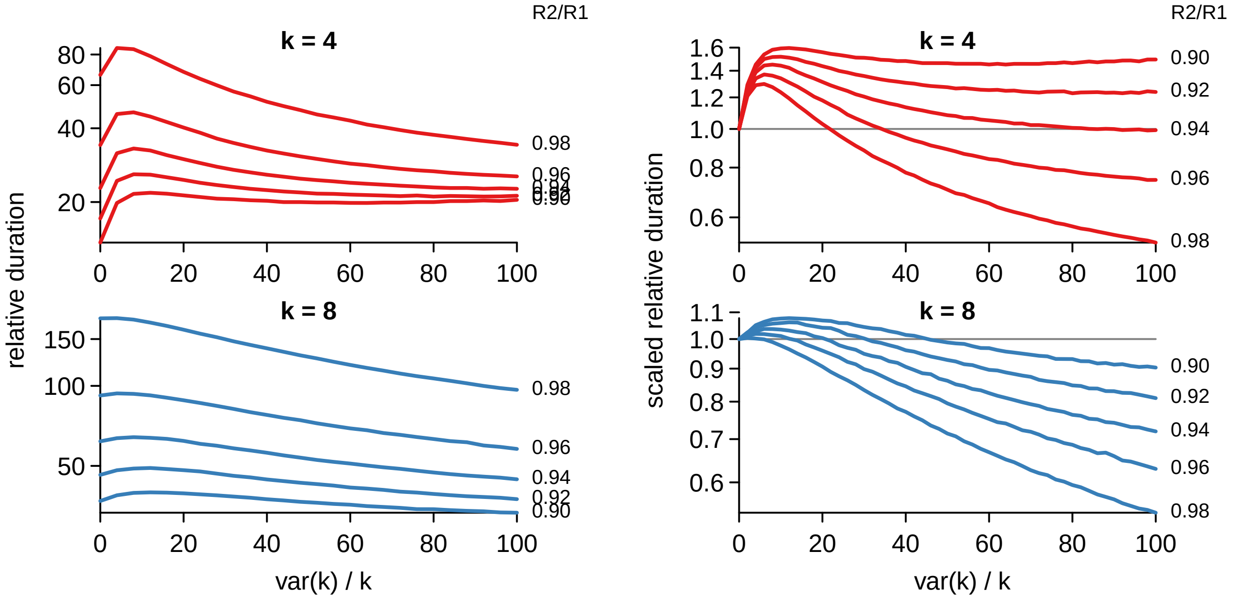

Figure S3: **Relative duration of competition versus initial spread.** The right panel shows the same data as in the left panel, though the curves are scaled to the relative duration at variance zero.

In order to see how higher order network structure might affect the spreading and competition phases, we compare the length of the competition phase to that of the initial spreading phase (Fig. S3). For the chosen parameter values, we see a confirmation that for small fitness differences, $0.9 < s < 1$, the competition phase is much longer than the initial spreading phase, up to 80 times longer for $k = 4$ and over 150 times longer for $k = 8$. Initially, as the degree variance of the network increases, so does the duration of the competition phase relative to the initial phase. However, for low degree networks ($k = 4$), the relative duration starts to decrease again above a certain variance ($var(k) \approx 10$). This is also observed for higher degree networks ($k = 8$), although the effect is lower in magnitude than for low degree networks.

Extinction rate of less fit strain. Beyond comparing the durations of the two phases, we can directly calculate the exponential decay rate of less fit strain during the competition phase. Since the decay appears to occur at a constant rate during the competition phase, we can calculate the rate close to the equilibrium point $(x_1^*, 0, y_1^*, 0, 0)$. Concentrating on the behaviour of x_2, we can rewrite the time derivative as

$$\frac{dx_2}{dt} = c\left(kR_2\frac{y_2}{x_2} - 1\right)x_2.$$

Thus, the prevalence of the less fit strain should decay with a rate,

$$r = c\left(kR_2\frac{y_2}{x_2} - 1\right).$$

In the limit $a \to \infty$ ($t \to \infty$),

$$\lim_{a\to\infty} r = \lim_{a\to\infty} c\left(kR_2\frac{dy_2/da}{dx_2/da} - 1\right).$$

Figure 4 shows that indeed the two limits converge.

Alternatively, the extinction rate can be computed numerically as the maximum eigenvalue of the Jacobian matrix calculated at the equilibrium point $(x_1^*, 0, y_1^*, 0, 0)$. Figure S5 shows the calculated decay rate together with rates extracted from simulations as a function of the degree variance. An increased degree variance accelerates the extinction dynamics of the less fit strain. Interestingly, the effect is strongest for low fitness differences.

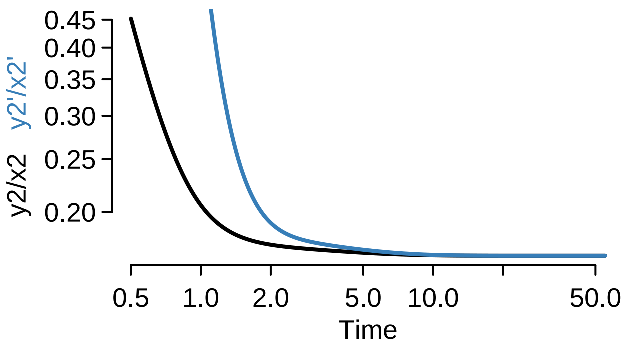

Figure S4: **Approximation of the ratio of the two strains using the time derivatives.**

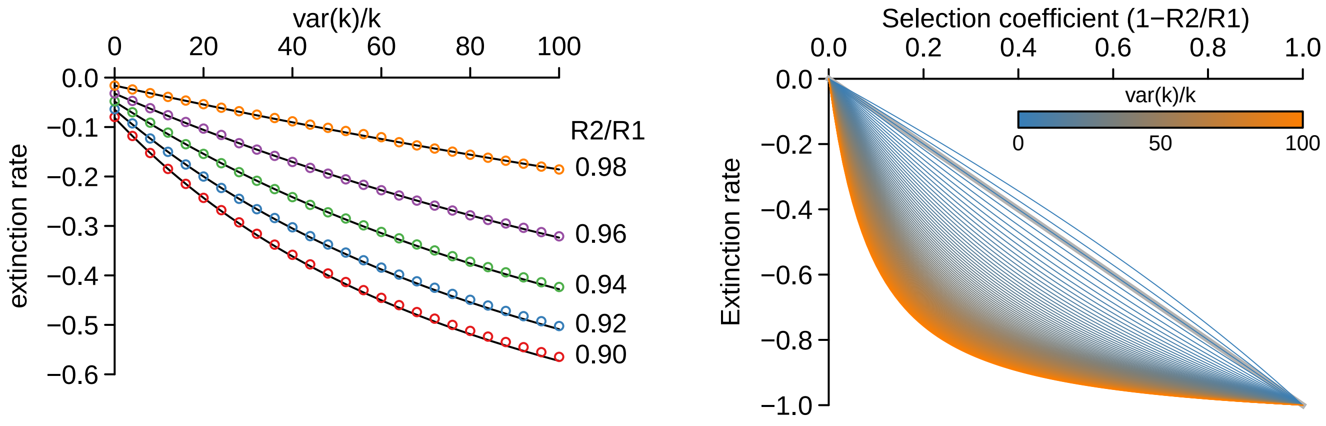

Figure S5: **Extinction rate of the less fit strain.** Left panel: Calculated decay rate (lines) and numerical approximations of the exponential decay (points) for different fitness differences. Right panel: Extinction rate as a function of the fitness difference for various degree variances.

References

[1] Keeling MJ, Rand DA, Morris AJ. Correlation models for childhood epidemics. Proceedings of the Royal Society of London. 1997;264(1385):1149–1156. doi:10.1098/rspb.1997.0159.

[2] Pastor-Satorras R, Vespignani A. Epidemic dynamics and endemic states in complex networks. Physical Review E. 2001;63(6):066117.
