# Supplementary material for: Host population structure impedes reversion to drug sensitivity after discontinuation of treatment
Source: PLoS Comput Biol. 2017 Aug 21;13(8):e1005704. doi: 10.1371/journal.pcbi.1005704 (PMC5602665; doi:10.1371/journal.pcbi.1005704)
Supplement: S2 Text — (PDF) [file pcbi.1005704.s006.pdf]

Host Population Structure Impedes Reversion to Drug Sensitivity After Discontinuation of Treatment:

Supporting Information

Jonas I Liechti[1,*], Gabriel E. Leventhal[1,2], Sebastian Bonhoeffer[1],

1 Institute for Integrative Biology, ETH Zürich, 8092 Zürich, Switzerland
2 Department of Civil and Environmental Engineering, Massachusetts Institute of Technology (MIT), Cambridge, MA, 02139, U.S.A.

* jonas.liechti@env.ethz.ch

Limit case death-birth process

It is relatively straightforward to show that in the limit of large transmission rates, the here used model tends exactly to the birth-death process on a network. For illustrative purposes we do not provide a general proof but demonstrate the exact mapping in the limit of large transmission rates for an exemplary microscopic configuration. It will become apparent that the presented approach can readily be adapted to any generalizations of the presented configuration and thus be used to build a general proof. This last step, however, is left for the interested reader.

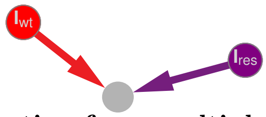

Figure S1: **Infection from multiple neighbours.**

Consider the situation from Figure 1 with 3 hosts connected in a line and the central node being susceptible. The birth-death process does not account for a susceptible state, and hence the middle node would immediately be infected by one of its neighbours. Which neighbour infects the middle node is determined by the relative fitness scores of the neighbours, $f_{wt} := 1$ resp. f_{mt}. The probability to be infected by the wild-type then writes:

$$P(I_{wt}) = \frac{1}{1 + f_{mt}}, \tag{1}$$

and by the mutant:

$$P(I_{mt}) = \frac{f_{mt}}{1 + f_{mt}}. \tag{2}$$

In the classical SIS case, however, the scenario from Figure 1 might occur. Further, several possibilities exist for the next event. Any of the infected nodes might recover or the middle node might get infected by any of its neighbours. For the sake of argument we assume that the middle node changed its status to susceptible at time $t = 0$ and we assess the probabilities of these potential next events at a specific time t^* after its state change.

The probability that at time t^* both infected nodes are still infected is simply given by:

$$P_{no-recovery}(t^*, \gamma) = (1 - (1 - e^{-\gamma t^*}))^2 = e^{-2\gamma t^*}. \tag{3}$$

Assuming that none of the neighbours recovered, the probability that at time t^* there has been an infection event for the middle node is simply given by:

$$P_{infection}(t^*, \beta_{wt} + \beta_{mt}) = 1 - e^{-(\beta_{wt}+\beta_{mt})t^*}, \tag{4}$$

and the probability that this infection was by the wild-type and mutant, respectively is:

$$P(I_{wt}) = \frac{\beta_{wt}}{\beta_{wt} + \beta_{mt}}, \tag{5}$$

$$P(I_{mt}) = \frac{\beta_{mt}}{\beta_{wt} + \beta_{mt}}. \tag{6}$$

What we would like to assess it the situation at time $t^* = 0$, so at the moment the middle node recovered, this in the limit of large transmission rates, in order to determine whether our SIS model is equivalent to a death-birth process.

We use the following Ansatz: Let $t^* = \frac{1}{\sqrt{\beta_{wt}}}$ and take the limit $\beta_{wt} \to \infty$ with $\frac{\beta_{mt}}{\beta_{wt}} = const$.

Clearly, $\lim_{\beta_{wt} \to \infty} \frac{1}{\sqrt{\beta_{wt}}} = 0$. Also, it is straightforward to show that in this limit the probability of any of the neighbours to recover tends to 0:

$$\lim_{\beta_{wt} \to \infty} P_{no-recovery}(\frac{1}{\sqrt{\beta_{wt}}}, \gamma) = \lim_{\beta_{wt} \to \infty} e^{-2\frac{\gamma}{\sqrt{\beta_{wt}}}} = 1. \tag{7}$$

For the probability of the middle node to become infected, we can use the constant relation between the transmission rates and write $\beta_{mt} = f_{mt} \cdot \beta_{wt}$. Equation 8 then becomes:

$$\lim_{\beta_{wt} \to \infty} P_{infection}(\frac{1}{\sqrt{\beta_{wt}}}, \beta_{wt}(1 + f_{mt})) = \lim_{\beta_{wt} \to \infty} 1 - e^{-\sqrt{\beta_{wt}}(1+f_{mt})} = 1. \tag{8}$$

This leaves us with a certain infection event: either infection of the middle node by the wild-type or by the mutant neighbour. If we rewrite Equations 5 and 6 we see that we exactly recover the death-birth case:

$$P(I_{wt}) = \frac{1}{1 + f_{mt}}, \tag{9}$$

$$P(I_{mt}) = \frac{f_{mt}}{1 + f_{mt}}. \tag{10}$$

This approach can easily be extended to a more general configuration with n wild-type and m mutant neighbours. This is, however, left for the interested reader.
