# Supplementary material for: Host population structure impedes reversion to drug sensitivity after discontinuation of treatment
Source: PLoS Comput Biol. 2017 Aug 21;13(8):e1005704. doi: 10.1371/journal.pcbi.1005704 (PMC5602665; doi:10.1371/journal.pcbi.1005704)
Supplement: S3 Text — (PDF) [file pcbi.1005704.s007.pdf]

Host Population Structure Impedes Reversion to Drug Sensitivity After Discontinuation of Treatment:

Supporting Information

Jonas I Liechti[1,*], Gabriel E. Leventhal[1,2], Sebastian Bonhoeffer[1],

1 Institute for Integrative Biology, ETH Zürich, 8092 Zürich, Switzerland
2 Department of Civil and Environmental Engineering, Massachusetts Institute of Technology (MIT), Cambridge, MA, 02139, U.S.A.

* jonas.liechti@env.ethz.ch

Impact of absolute prevalence at treatment stop

In this study we treat the variance in the degree distribution as a control parameter we set in order to report the direct correlation between degree variance and reversion. This approach allows us to determine the effect the degree variance has on the probability of reversion. It cannot, however, determine whether the variance mechanistically acts directly, via the prevalence, or via another property on the probability of reversion. While our main conclusions are based on the correlations of degree variance and the probability of reversion and thus indifferent to the specific mode of action we would like to exclude that the probability of reversion is merely determined as the results of a "numbers game", but actually is a result of the altered dynamical properties when changing variance. To do so, we investigate whether the prevalence at treatment stop has a direct effect of the probability of reversion.

Note that at treatment start the wild-type strain is, by design, in a quasi steady state. During treatment, however, neither the wild-type nor the resistant strain are ever in a quasi steady state. This is because both treatment and competition would eventually lead to extinction of the wild-type strain (if drug pressure is sufficient), after which the resistant strain might reach a quasi steady state. As we stop the treatment phase at a specific fraction of resistant infecteds, this scenario never occurs. Nevertheless, we can assess whether the absolute prevalence at treatment stop affects the probability of reversion. Given that at treatment halt we are not in a quasi steady state and that the condition for a treatment halt is based on the relative frequency of resistant infecteds, the total number of infecteds might vary from case to case. To show that this is indeed the case, Fig 1 shows an exemplary distribution of absolute prevalence of infecteds at treatment halt. If the

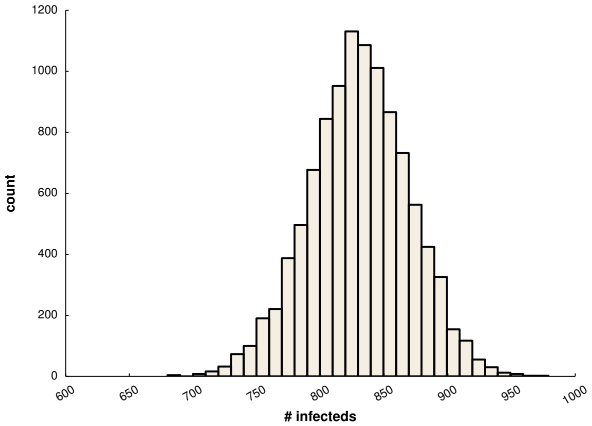

Figure S1: **Distribution of absolute prevalence at treatment stop**. Shown is an ensemble of 10521 simulations with a host structure of size $N = 2000$, zero variance in degree and equal fitness of both strains in the absence of treatment.

absolute prevalence influences the reversion probability, we should see a difference in reversion probability when comparing the sub-ensembles of both tails from this distribution. As the parameters for these sub-ensembles are identical, we can rule out any cross correlations of network heterogeneity with both prevalence and reversion probability. Figure 2 considers the differences in absolute prevalence at treatment stop and the resulting effect

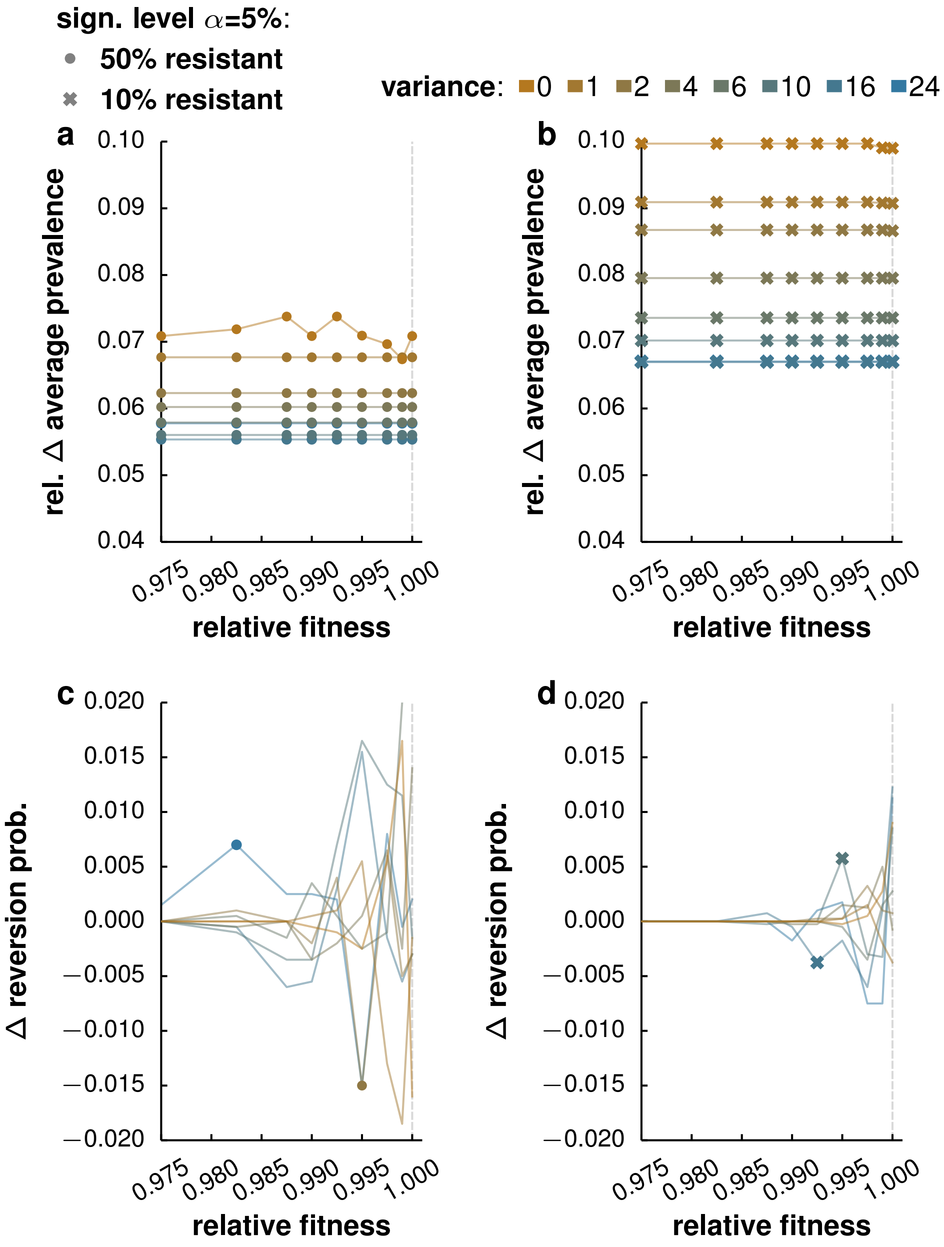

Figure S2: **Difference in absolute prevalence and reversion probability between the tails of the distribution of number of infected individuals at treatment stop**. Marker correspond to significant deviations at a significance level of 5%.

on the reversion probability between two sub-ensembles (both tails) for various network heterogeneities and, in the left column (panel a and c) a relative fraction of resistant infecteds of 0.5 and, in the right column (panels b and d), a relative fraction of 0.1. For the left column, the size of each sub-sample is 2000 simulations and for the right column, as we ran more simulations for this scenario, 4000. Panels a and b indicate the relative difference in absolute prevalence between those two sub-samples for all parameter combinations. Marked points indicate a significant deviation from the hypothesis of equal mean in absolute prevalence between the two tails (standard two sample t-test with significance level of $\alpha = 5\%$). Panels c and d report the difference in probability of reversion between the two tails for the two cases. To test whether the reversion probability is different for the two sub-samples we applied an exact Fischer test for all variances and fitness values independently with the hypothesis that the probability of reversion is the same in both sub-samples. Marked points indicate a significant deviation from this hypothesis for a significance level of $\alpha = 5\%$. For both fraction of resistants, i.e. 10% and 50%, among the resulting 72 differences only 2 were significant. Thus, while we observe only significant differences in absolute prevalence at treatment stop ranging from 5% to 10%, there is no statistical evidence these variations affect the probability of reversion. While this is no proof that the probability of reversion is

independent on the absolute prevalence at treatment stop, it shows that, at least in our simulations, there is no evidence for a direct relation between these two quantities.
